# Experimental Study on Single Corner Cold Bending Mechanical Response of Laminated of PVB Interlayer Tempered Glass Panes and the Coupling Effect with Load

**DOI:** 10.3390/ma14226914

**Published:** 2021-11-16

**Authors:** Xide Zhang, Wei Zhou, Mingcai Xu

**Affiliations:** 1 College of Civil Engineering and Architecture, Guangxi University, Nanning 530004, China; 2 Key Laboratory of Engineering Disaster Prevention and Structure Safety of Ministry of Education, Guangxi University, Nanning 530004, China; 3 Guangxi Key Laboratory of Disaster Prevention and Mitigation and Engineering Safety, Guangxi University, Nanning 530004, China

**Keywords:** laminated tempered glass, cold bending, mechanical response, coupling effect, experimental study

## Abstract

The cold bending method is a type of curved glass curtain wall construction method that has been used in practical engineering for a short time. It has the advantages of simple operation, high efficiency and low cost. However, the mechanical response and properties of glass panes caused by cold bending have not been solved effectively. To study the mechanical response and the properties of cold formed laminated tempered glass panes after applying with a wind load, cold bending and load tests of 9 laminated tempered glass panes were conducted by the orthogonal experimental design method. The effects of cold bending curvature, glass pane thickness and interlayer thickness were considered. In this paper, the response law of cold bending stress to the curvature and the relationship among the influencing factors were analyzed. The variation process of stress, the deflection of cold-formed glass panes under uniform load and the characteristics affected by cold-formed stress and deformation were studied. The results show that the cold bending stress is distributed in a saddle shape, and the curvature has the greatest influence on the cold bending stress, followed by the thickness of the glass panes. The influence of the interlayer thickness is small. The maximum stress appears near the corner of the short side direction adjacent to the cold bending corner. The cold bending stress increases linearly with increasing cold bending curvature. The cold bending stress and deformation have little effect on the change process of the later stage load effect.

## 1. Introduction

Traditionally, there are two main methods for the construction of curved glass curtain walls. One of the methods is plane fitting, which divides the whole curved surface into small plane plate units and synthesizes the required surface effect through splicing between units. This method is economical, but it has a poor artistic effect. The other is to divide the curtain wall into regular glass panes and then form it by hot bending according to the surface shape in the factory. This method has a good construction effect, but the required process is complex, and the cost is much higher than that of the first method. In particular, a curtain wall with a complex and extremely irregular curved surface can require each glass pane to make a hot bending mold alone. The molds made are generally not reusable, which results in a large increase in the construction cost and difficulty. Therefore, in recent years, a cold bending method has emerged in the construction of glass curtain walls that have complex and irregular surfaces [1]. The cold bending forming method utilizes the deformation ability of glass, fixing the glass with support to form a cold bend. During the construction, the support system is installed according to the shape of the curved surface of the curtain wall, and then, a certain force is applied to the plane glass panes to the position, and it is fixed to form the designed surface. This cold bending forming method is easy to operate, inexpensive, and can greatly shorten the construction period. Therefore, it is gradually welcomed by construction units around the world.

Due to its significant advantages, the cold bending method has been applied in large urban landscape public buildings such as museums, airport terminal buildings and exhibition centers in recent years. There are representative buildings, such as the Shining Towers in Abu Dhabi [2], the Credit Libanais bank in Lebanon [3], the Seele Glass Bridge in Germany [4], the Strasbourg Railway Station in Alsace, France [5], the new roof structure of the Amsterdam bus station [6], the Wujiang Shengze International Convention and Exhibition Center in China, a project in Shanghai Hongqiao [7], and Suzhou Central Plaza [8]. Especially for the new terminal building at Nanning Wuxu International Airport, the hyperbolic glass curtain wall designed by the cold bending method has a good effect, saving the cost, shortening the construction and achieving significant economic benefits.

It is obvious that the glass pane will produce a corresponding mechanical response after cold bending and will produce cold bending stress in its interior. The stress will not disappear after the glass pane is installed in place. The coupling effect of cold bending stress and external load belongs to the stress problem of thin plates with primary stress, and the primary stress or initial deflection affects the mechanical properties of the glass pane [9]. The displacement generated by cold bending and loading is generally much larger than the thickness of the glass pane, and it has obvious geometric nonlinear characteristics. The mechanical problem is complex. Therefore, the calculation method of the bearing capacity of cold-formed glass panes must be reestablished.

Under the applications of practical engineering, researchers began to study the stress of cold-formed glass panes. Laura Galuppi et al. [10,11,12] studied the failure limit of unidirectional cold-formed laminated glass panes, the distribution of interlaminar shear stress and the influence of the cold bending shape and conducted a unidirectional cold bending experiment. Kyriaki Corinna Datsiou [13] studied the cold bending limit of a single glass pane by experimental and numerical simulations. Andrea Spagnoli [14] simulated cold bending of glass with aluminum plates of similar material properties and finite element simulation. Felix Nicklisch et al. [15] conducted a single corner warping cold bending test and a numerical study of single glass on a wooden frame, which proved that the bending stress of a glass pane is very sensitive to the supporting conditions of the glass edge. Jan Belis [16] have been determined the major influencing factors in the present research of a cold bending process on a circular bending mould with a radius of three metres. Pottmann Helmut [17] conducted large double-curvature glazing of any form can be approximated by cylindrically bent panels. There are relatively few studies in China. Tang Jiyu et al. [1] conducted a confirmatory cold bending test on glass panes of the new terminal building of Nanning Wuxu International Airport. Sun Jian and Jin Zhiqiang [7,18] verified the reliability, fatigue performance and construction technology of a single cold-formed glass pane of a large bird-shaped lighting roof in Suzhou Central Plaza through experiments. Xide Zhang [19] conducted an experimental study on cold bending and coupling with a uniform load of hollow tempered glass panes.

Although some researchers have performed relevant research work [4,5,6,10,11,12,13,16], it is still in the initial stage overall. There are a few types of glass studies, which are limited to single glass [4,13,16] and a small amount of laminated glass [5,11,12,16]. The mechanical response of multilayer composite glass panes to cold bending is not understood, and there are few studies on the coupling effect of cold bending and loading. At present, there are only a small number of engineering application examples and targeted test reports in the published papers. Most of these papers only reflect a specific engineering case, and there are few research achievements in the basic aspects of application. Up to now, the design method of cold bending glass has not been included in the current specification, so it can be seen that the research work fails to keep up with the needs of practical engineering, which greatly restricts the development of cold bending forming method. For engineering projects completed by this method, this reliability cannot be guaranteed, and there can be unpredictable security risks. Therefore, it is of great significance to solve the bearing capacity problem of cold-formed glass panes as soon as possible for the application of cold bending method.

Because the mechanical problem of thin plates with geometrical nonlinearity and initial stress is complicated in theory, the mechanical state and boundary conditions of cold-formed glass panes have fundamentally changed compared with plane glass panes, and the theoretical derivation and expression could be very complicated. Therefore, to meet the needs of engineering practice as soon as possible, it is more appropriate to explore the mechanical problems of cold-formed laminated glass panes through experimental research. Since the direction of load action is different from the positive direction of normal vector on the upper surface of glass panes surface, its effect is different. But the most unfavorable condition is that the load direction is opposite to the concave direction of the glass pane, which is called reverse coupling. In this paper, laminated tempered glass panes commonly used in glass curtain walls are taken as the research object. The distribution law of cold bending stress and its change process, as well as the effect of cold bending stress and load coupling, are studied through experiments. The influence of the cold bending curvature, glass plate thickness and laminated thickness is considered in the experiment to provide a reference for engineering design and the compilation of relevant specifications.

This paper focuses on the reverse mechanical properties of cold formed laminated glass. Through the experimental research combined with numerical simulation, some beneficial conclusions are obtained. At the same time, through the most adverse combination of various factors to the design of the cold formed laminated glass curtain wall put forward more safety requirements. In this paper, the reverse mechanical properties of cold-formed laminated glass are studied and design suggestions are put forward, which fills in the blank of mechanical behavior research of cold-formed glass structure to a certain extent. Combined with the mechanical research of cold-formed glass above by our research group, the experimental data and research materials are provided for future standard revision.

## 2. Experiments

### 2.1. Specimen Design

According to the structure of laminated glass, the cold bending curvature *β*, glass thickness t and interlayer thickness *t*_v_ were selected as the influencing factors of the test. The test scheme was designed according to the orthogonal experimental design method of three factors and three levels. A total of 9 specimens were required. The plane size of the glass pane was 2200 mm × 1200 mm. The thickness of the single glass pane was 5 mm, 6 mm and 8 mm, which are commonly used in the project. The thicknesses of the upper and lower two glass plates were the same. The thickness of the interlayer was 1.14 mm, 1.52 mm and 2.28 mm according to the commonly used production specifications. The glass was domestic standard tempered glass, which is made of ordinary flat glass composed of Na2O·CaO·6SiO2 after processing. And the interlayer material was polyvinyl butyral (PVB), which is commonly used. The specimens were made by professional manufacturers according to Chinese production standards.

According to the concept of sheet warping, the curvature index of single corner cold bending is defined as [1]

(1) $$\beta =\frac{s}{2c}\times 100\%$$

**Figure 1:**
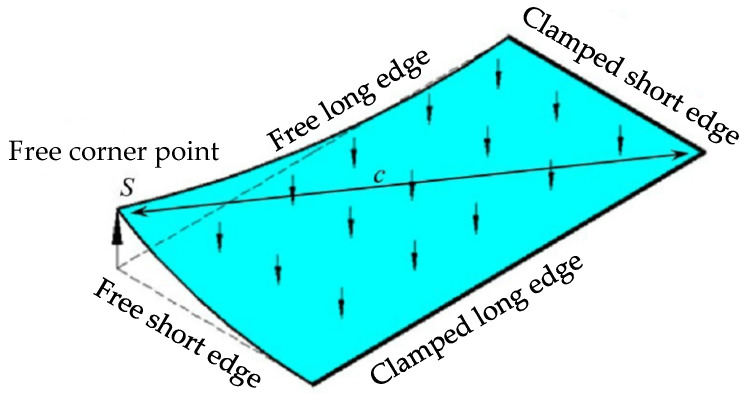
Cold bending and load action mode.

In this formula, *β* is the cold bending curvature, *s* is the maximum cold bending displacement of the free corner, and *c* is the diagonal length of the glass plate, as shown in Figure 1.

In the cold bending test, the maximum curvature *β*_max_ is 0%, 0.6% and 1.2%. A curvature of 0% is used to compare the performance with or without cold bending. When *β*_max_ is 0.6% and 1.2%, the maximum displacement of the free corner point is 30 mm and 60 mm, respectively. The specific parameters of each specimen are shown in Table 1.

### 2.2. Experimental Settings

**Figure 2:**
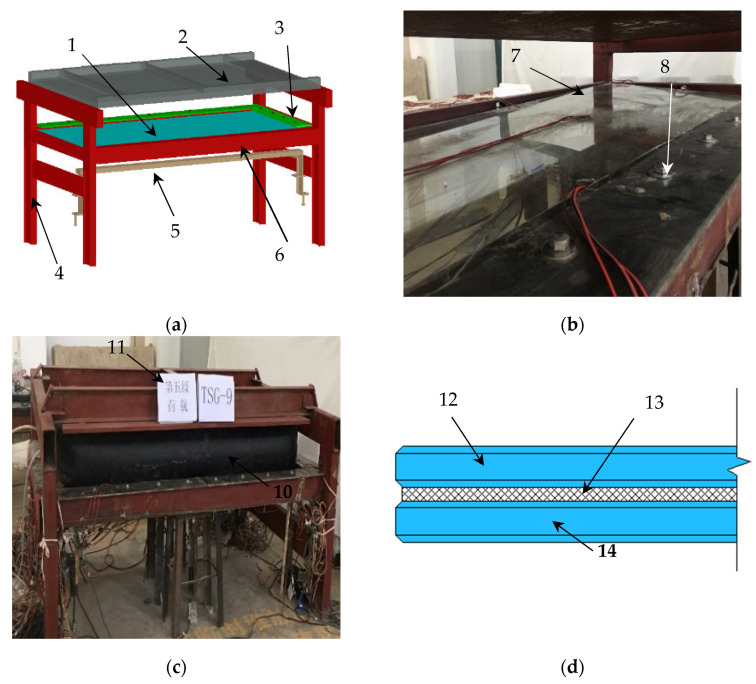
(**a**) Test device; (**b**) Cold bending test; (**c**) Load test; (**d**) The view of the section of the pane. The symbols in the figures stand for 1. glass, 2. counterforce frame, 3. glass fixed devices, 4. steel set, 5. cold bending loading device, 6. cold bending forced in-place loader, 7. cold bending point, 8. glass fixed devices, 9. counterforce frame, and 10. Airbag, 11. Grade 5 load, 12. the tempered glass on top, 13. PVB interlayer, 14. the tempered glass underneath.

To simulate the working state of the glass panes in practical engineering, a set of test devices is specially designed and manufactured, as shown in Figure 2a, which can realize cold bending and uniform load testing of frame-supported glass panes. The test device is composed of a steel bracket, glass plate supporting frame, glass fixing device, load reaction frame and cold bending forced in-place loader. A forced in-place loader has been used for cold bending construction and testing of glass curtain walls at Nanning Wuxu International Airport Terminal [1]. A pair of adjacent edges of the supporting frame maintain the same plane as the fixed edge of the glass pane and the other pair of adjacent edges maintain the same plane as the free edge. The intersection corner point is called the free corner point, and the position of the free corner point and the free edge can be adjusted to realize the testing of different cold bending curvatures. A plane structure is set up above the device as the reaction frame in the load test, and an airbag is installed between the counterforce frame and the glass panes to simulate the uniform load. The test site is shown in Figure 2b,c. The view of the section of the laminated of PVB interlayer tempered glass pane is shown in Figure 2d.

The test of each specimen is divided into two stages. The first stage is the cold bending test. This stage is mainly to study the stress distribution and change process of the laminated glass panes in the cold bending process. In the cold bending test, the glass plate specimen is smoothly placed on the supporting frame and then fixed with a fixed device at the clamped edge. In the free long edge, the cold bending loading device is installed to conduct the cold bending test of the glass plate. To study the stress change process and the influence of different cold bending degrees in the cold bending process, the cold bending displacement is applied according to the cold bending curvature graded 0.20%. In other words, when the maximum cold bending curvature is 0.6%, the cold bending curvature is loaded by three levels: 0.2%, 0.4%, and 0.6%. When the maximum cold bending curvature is 1.2%, the cold bending curvature is loaded by 0.2%, 0.4%, 0.6%, 0.8%, 1.0% and 1.2%. The displacement of each level stays for 15 min, and then, the static strain gauge is used to read the strain and displacement at the same time. The cold bending displacement is measured by the displacement sensor installed below the free corner point. After the cold bending loading is completed, the two free edges are fixed according to the same method as the fixed edge.

**Figure 3:**
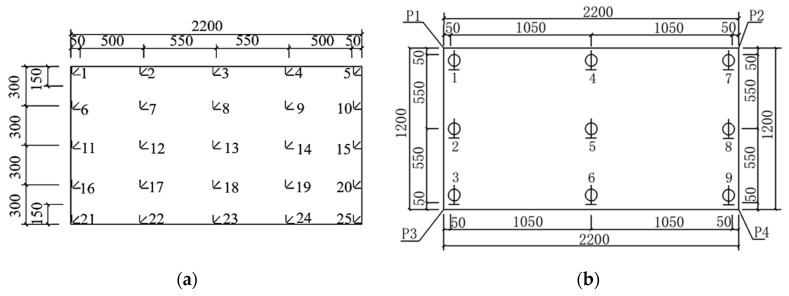
(**a**) Arrangement of strain measuring points on the lower surface of the glass; (**b**) Arrangement of deflection measuring points on the lower surface of the glass.

After the cold bending test was completed, the load test in the second stage was conducted to study the coupling effect of the normal load and cold bending stress. The airbag is installed between the glass pane and the counterforce frame to simulate the static action of a uniform wind load. As shown in Figure 3, the load direction is opposite to the normal direction of the glass pane surface, in other words, the glass curtain wall is concave in shape. In this state, the tensile stress generated by the cold bending of the glass pane and the maximum tensile stress generated by the load are superimposed in the same direction. In mechanics, it is the most unfavorable stress condition of the glass panes of the curtain wall, which is called the reverse coupling effect. According to reference [19], the maximum wind pressure of the outer glass is grade 9, and the corresponding load is 5 kPa. The load test is conducted in five stages. Each stage is loaded with 1 kPa, and each stage is loaded for 15 min. The loading value is measured by the gas pressure gauge installed on the air pump. The specimen with 0% cold bending curvature does not conduct a cold bending test, and the load test in the second stage is directly conducted. Repeated tests were performed twice after each specimen test to reduce errors.

### 2.3. Measuring Point Arrangement

Since glass is a brittle elastic material and the compressive strength is much higher than the tensile strength, its bearing capacity depends mainly on the maximum tensile strength of the glass. Therefore, the tensile stress is mainly measured in the test. Figure 1 shows that the maximum tensile stress should occur on the lower surface of the glass pane during cold bending and load testing. Therefore, 25 strain measuring points are evenly arranged on the lower surface. The bearing capacity of each part of the glass pane is different. The stress standard values of the edge and end face of the glass panes are smaller than those of the middle [1]. Therefore, when the measuring points are arranged, the measuring points on the edge should be as close as possible to the edge. The arrangement and number of each measuring point are shown in Figure 3a. The relative position of each measuring point is from the bottom to the top when looking up. Since the principal stress direction of the cold bending distortion surface is unknown, all of the strain measuring points adopt the strain rosette. A displacement sensor is arranged on the lower surface of the glass pane, and the position is the same as the strain measuring point on the upper surface. It is also located at the center of the glass pane, the four corners and the midpoint of the four sides. There are nine displacement measuring points. As shown in Figure 3b, all of the measuring points are the same in the cold bending test and load test.

## 3. Cold Bending Test Results and Analysis

### 3.1. Response of Cold Bending Stress to Cold Bending Curvature

**Figure 4:**
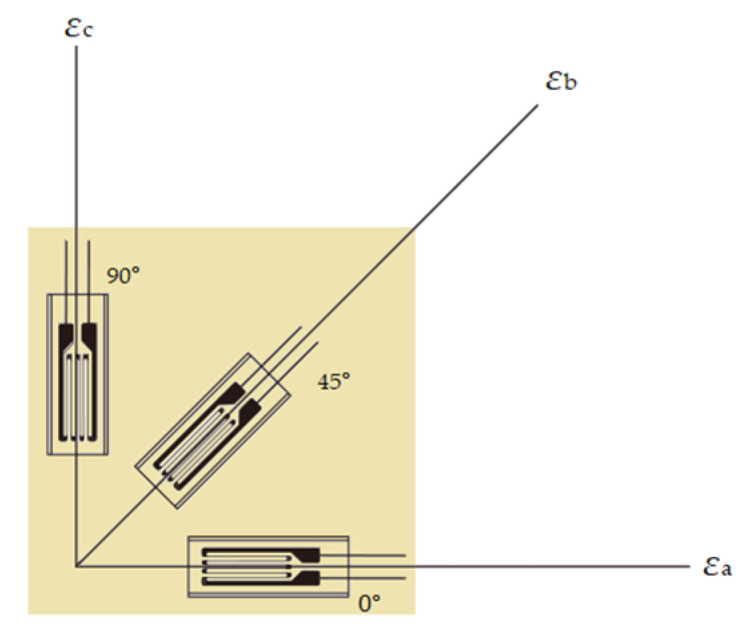
The strain rosette.

According to the strain measurement results, the maximum principal tensile stress of each measuring point can be obtained from Equations (2) and (3) through the three strain values measured by the strain rosette shown in the Figure 4. The elastic modulus E of the glass is 72 × 10^3^ MPa, and Poisson′s ratio *μ* is 0.2. Since the thickness of the glass plate is smaller than its size, the influence of the out-of-plane direction is not considered.

(2) $$\begin{array}{c} \varepsilon_{max}\\ \varepsilon_{min} \end{array}=\frac{\varepsilon_{a}+\varepsilon_{c}}{2}\pm \frac{1}{2}\sqrt{{\left(\varepsilon_{a}-\varepsilon_{c}\right)}^{2}+{\left(\varepsilon_{a}+\varepsilon_{b}-\varepsilon_{c}\right)}^{2}}$$

(3) $$\begin{array}{c} \sigma_{max}\\ \sigma_{min} \end{array}=\frac{E}{2}\left[\frac{\varepsilon_{a}+\varepsilon_{c}}{1-\mu }\pm \sqrt{{\left(\varepsilon_{a}-\varepsilon_{c}\right)}^{2}+{\left(\varepsilon_{a}+\varepsilon_{b}-\varepsilon_{c}\right)}^{2}}\right]$$

**Figure 5:**
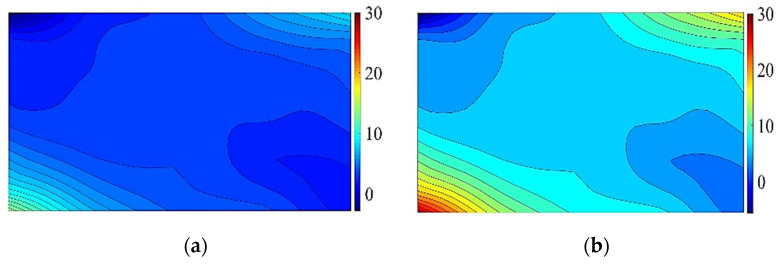
The contour plots of cold bending principal tensile stress of TSG-3. (**a**) *β* = 0.6%; (**b**) *β* = 1.2%.

As mentioned above, the free corner point bends upward, and the maximum principal tensile stress is located on the lower surface of the glass pane. To analyze the distribution of the principal tensile stress on the lower surface during the cold bending process, according to the data of individual test points, the corresponding main tensile stress contour plots are drawn by the method similar to drawing contour lines. Figure 5 shows the main tensile stress contour plots of the lower surface of TSG-3 when the cold bending rates are 0.6% and 1.2%, and the contour plot shapes of the other specimens are basically the same. Figure 5 shows the maximum principal tensile stress contour plot of the lower surface of TSG-3 when the cold bending curvature is 0.6% and 1.2%, and the contour plots of the other specimens are basically the same. It can be seen from the figure that the maximum principal tensile stress occurs at the intersection of the free short edge and the fixed long edge at the No. 21 measuring point in the lower-left corner, followed by the intersection of the free long edge and the fixed short edge at the No. 5 measuring point. This finding is due to the same cold bending displacement; the curvature radius of the short edge is smaller than that of the long edge, and the curvature is larger, resulting in a larger cold bending stress, and the corresponding corner points of the cold bending angle and its diagonal are the smallest. A certain cold bending tensile stress is also produced near the center point of the pane. On the whole, there is a saddle-shaped stress distribution with the diagonal as the axis is formed. Comparing the two figures, it can be seen that the cold bending curvature has a large influence on the cold bending stress. Since the edge bearing capacity of the glass panes is approximately 20% smaller than that of the middle of the plate [1], the distribution of the cold bending stress is unfavorable to the stress of the glass panes.

**Figure 6:**
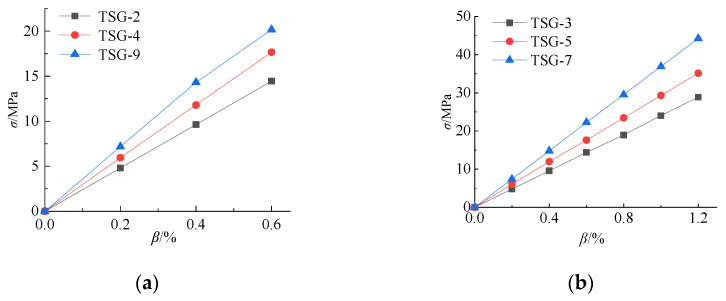
Relationship between cold bending stress and cold bending curvature of NO.21. (**a**) *β* = 0.6%; (**b**) *β* = 1.2%.

**Figure 7:**
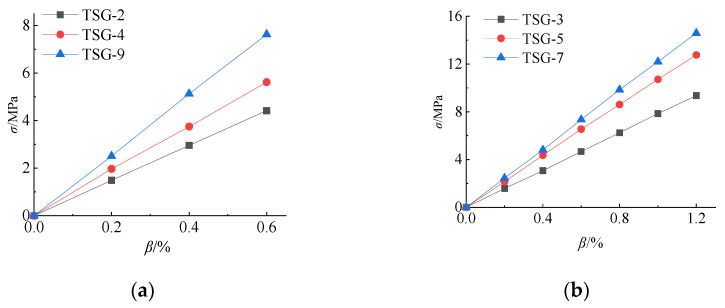
Relationship between cold bending stress and cold bending curvature of NO.13. (**a**) *β* = 0.6%; (**b**) *β* = 1.2%.

To analyze the response process of the cold bending stress to the cold bending curvature, the relationship curves between the cold bending stress and the cold bending curvature of measuring point No. 21 and measuring point No. 13 in the center of the glass plate are drawn, as shown in Figure 6 and Figure 7. From these figures, it can be seen that the cold bending stress of the two measuring points increases linearly with increasing cold bending curvature. The relationship curve of thick, large glass is located at the top of the figure, which indicates that the stress growth rate of the thick, large glass is faster. The greater the thickness of the glass panes, the greater the bending stiffness of the panes, and the greater the cold bending stress.

### 3.2. Analysis of the Influencing Factors for Cold Bending Stress

When the orthogonal experimental design is adopted in the test, the influence law of each factor must be determined by range analysis. The maximum principal tensile stress *σ*_m_ at measuring points No. 13 and No. 21 is taken as the analysis index. The test results of nine specimens are shown in Table 2. Among them, TSG-1, TSG-6 and TSG-8 are noncold-bending specimens, and the cold bending stress is directly taken as 0. The range analysis of the maximum stress *σ*_m_ in Table 2 is conducted. The results are shown in Table 3 and Table 4. In these tables, *K_i_* represents the average stress of the influencing factor at level *i*. Each factor in this test has three horizontal values, and *i* is 1, 2 and 3 according to the horizontal value from low to high. For example, the test horizontal values of the influencing factor’s cold bending curvature *β* are 0%, 0.6% and 1.2%, respectively. When calculating the *K*_1_ of t = 5 mm at measuring point No. 21, the size of *K*_1_ can be obtained as 14.43 MPa from the stress test values of TSG-1, TSG-2 and TSG-3 at measuring point No. 21 with a thickness is 5 mm in Table 2. When calculating the *K*_1_ of *t*_v_ = 1.14 mm at measuring point No. 21, the size of *K*_1_ can be obtained as 20.64 MPa from the stress test values of TSG-1, TSG-4 and TSG-7 at measuring point No. 21 with the interlayer thickness is 1.14 mm in Table 2. When calculating the *K*_2_ of *β* = 0.6% at measuring point No. 21, the size of *K*_2_ can be obtained as 17.09 MPa from the stress test values of TSG-2, TSG-4 and TSG-9 at measuring point No. 21 with a cold bending curvature of 0.6% in Table 2. The results of the range analysis of the cold bending stress at measuring point No. 21 are shown in Table 3. The range *R* = ǀ*K*_3_-*K*_1_ǀ reflects the degree of change of the analysis index when the influencing factors change from 1 to 3 at the test level. The greater the value of *R* is, the greater the influence of the factor on the analysis index. Therefore, the primary and secondary relationship of the influencing factors can be judged according to the size of *R*. It can be seen from Table 3 and Table 4 that the cold bending curvature *β* has the greatest influence on the cold bending stress, followed by the thickness of the glass panes t and the thickness of the PVB interlayer film *t*_v_. Comparing the range of the glass pane thickness and the PVB interlayer film thickness with the range of the cold bending curvature, the range ratio is obtained. The influence of the glass plate thickness is approximately 20% of the cold bending curvature, and the influence of the PVB interlayer film thickness is approximately 10% of the cold bending curvature. The influence of the interlayer should be considered when the cold bending stress is unfavorable to the bearing capacity.

**Figure 8:**
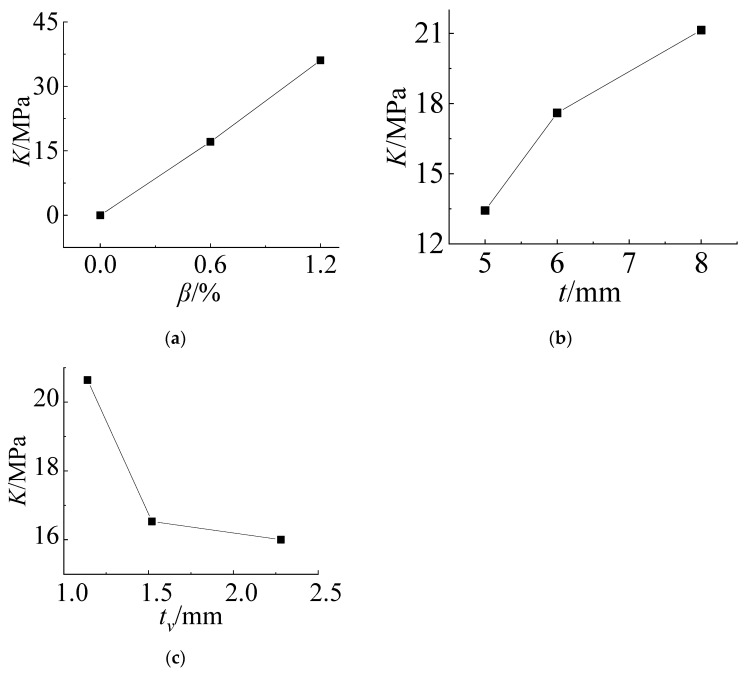
Influence of the test factors on the cold bending stress of No. 21. (**a**) *β*~*K*; (**b**) t~*K*; (**c**) *t*_v_~*K*.

According to the data of Table 3, the relationship between the *K* value of each influencing factor and its horizontal value at the No. 21 measuring point can be drawn, as shown in Figure 8. From Figure 8a, it can be seen that the cold bending stress increases with increasing cold bending curvature and basically shows a linear trend. Figure 8b shows that the cold bending stress increases with increasing thickness of the glass panes because the greater the thickness is, the greater the stiffness is, but it is nonlinear. With increasing thickness, the stress growth tends to be flat. Figure 8c shows that the cold bending stress value decreases with increasing thickness of the PVB interlayer, and the rate of decrease is fast at first and then slow. The interlayer connects the upper and lower glass panes and withstands the shear effect parallel to the glass pane in the cold bending process. Since the PVB adhesive is a viscoelastic material with low strength, the greater the thickness is, the weaker the shear-bearing capacity of the glass panes in-plane; additionally, the weaker the ability to transfer shear stress is, and the smaller the contribution to the overall bending stiffness of the laminated glass panes. The variation trends of the stress at measuring points No. 13 and No. 21 are basically the same under the same factors.

## 4. Load Stress Test Results and Analysis

### 4.1. Coupling Characteristics of the Load Stress and the Cold Bending Stress

Since the direction of the main tensile stress generated by cold bending was different from that generated by the load, the principal tensile stress obtained by uniformly distributed load test and the principal tensile stress generated by cold bending test are transformed into the same coordinate system for superposition. The coordinate transformation method of stress is shown in Equations (4)–(6). *θ* is the included Angle between *Ox* axis in the maximum principal stress coordinate system *Oxy* of uniformly distributed load test and *Ox′* axis in the maximum principal stress coordinate system *Ox′y′* of cold bending test. Since the thickness of the glass panes is smaller than its size, the influence of the out-of-plane direction is not considered, the superposition method was conducted according to the vector superposition principle, and the total stress *σ*_mp_ after the coupling of the glass cold bending stress and the uniform load was obtained. When the maximum load is 5 kPa, the maximum principal tensile stress after coupling the load stress and cold bending stress at measuring points No. 13 and No. 21 are listed in Table 5. It can be seen from the table that the stress of each specimen at measuring point No. 13 is greater than that at measuring point No. 21 except for TSG-7. The test parameters of TSG-7 for the cold bending curvature and the thickness of the glass pane are large, while the thickness of the PVB film interlayer is small. From the analysis of 3.2, it can be seen that the cold bending stress generated by such parameters is generated at the edge of the glass panes. Considering that the strength standard value of the glass panes at the edge is small [1], the design of the cold bending glass panes should attempt to avoid the situation in which the bearing capacity is controlled by cold bending stress.

(4) $$\sigma_{x^{\prime }}=\sigma_{x}\cos^{2}\theta +\sigma_{y}\sin^{2}\theta -2\tau_{xy}\cos\theta \sin\theta$$

(5) $$\sigma_{y^{\prime }}=\sigma_{x}\sin^{2}\theta +\sigma_{y}\cos^{2}\theta +2\tau_{xy}\cos\theta \sin\theta$$

(6) $$\tau_{xy^{'}}=\;\left(\sigma_{x}-\sigma_{y}\right)\cos\theta \sin\theta +\tau_{xy}\left(\cos^{2}\theta -\sin^{2}\theta \right)$$

According to the data in Table 5, the range analysis of the factors that affect the maximum principal tensile stress of the load can be conducted according to the method of 3.2. The range analysis results of measuring point No. 13 in the center of the pane are shown in Table 6. It can be seen from the table that the cold bending curvature is the most influential factor because the cold bending curvature is the first factor that affects the cold bending stress, and the cold bending stress is the initial stress of the load test. Therefore, its influence is also the largest, followed by the influence of the thickness of the glass panes, and the range is 47.2% of the cold bending curvature. The influence of the interlayer thickness is the smallest, only 3.32% of the cold bending curvature. When the interlayer thickness is from 1.14 mm to 1.52 mm, the stress *K* value decreases by 0.28 MPa, and when the interlayer thickness is from 1.52 mm to 2.28 mm, it decreases by 0.22 MPa. Therefore, the thickness of the PVB interlayer has little effect on the cold bending coupling stress of the load, and its influence can be ignored when it is beneficial to the bearing capacity.

**Figure 9:**
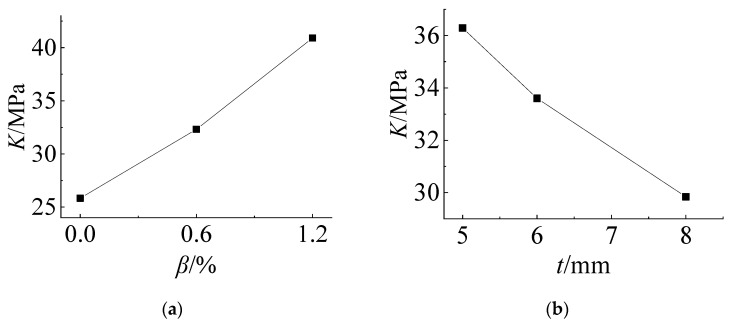

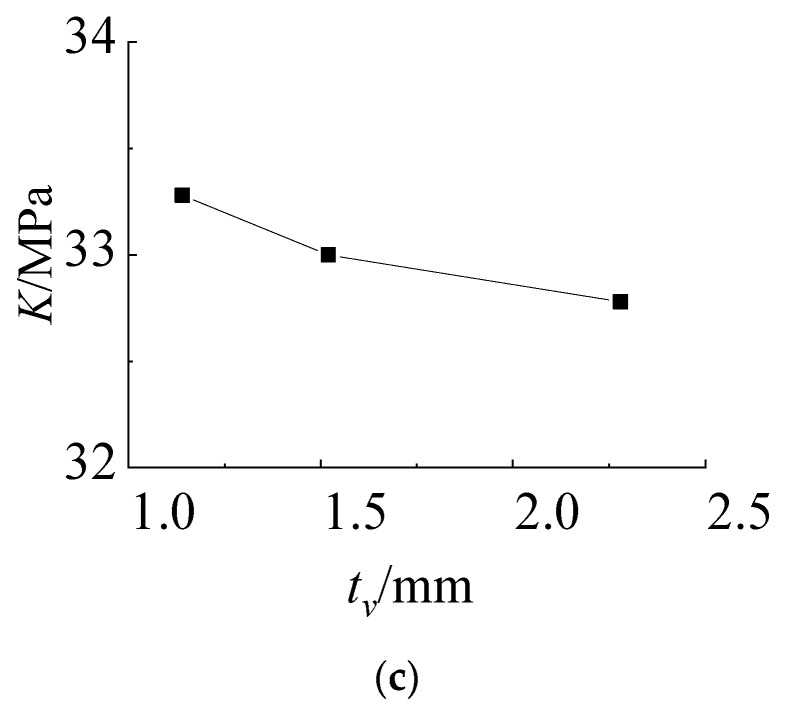
Influence of the test factors on the load stress of No. 13. (**a**) *β*~*K*; (**b**) t~*K*; (**c**) *t*_v_~*K*.

**Figure 10:**
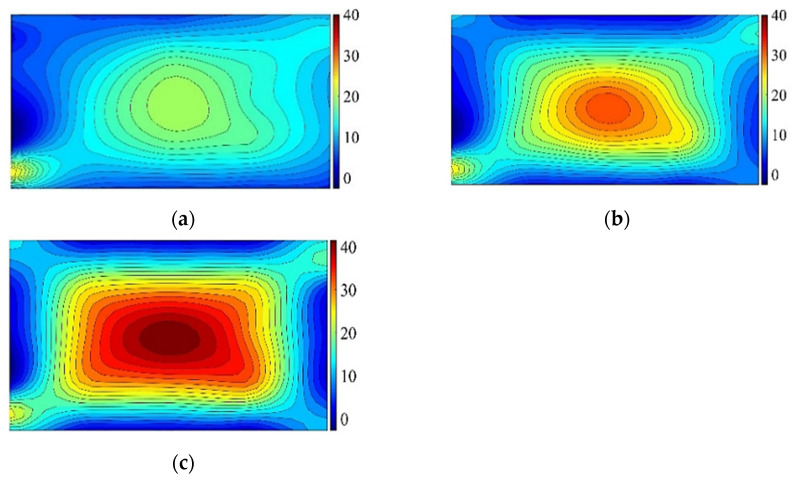
Cold bending and load coupled stress contour plots of TSG-3. (**a**) 1 kPa; (**b**) 3 kPa; (**c**) 5 kPa.

**Figure 11:**
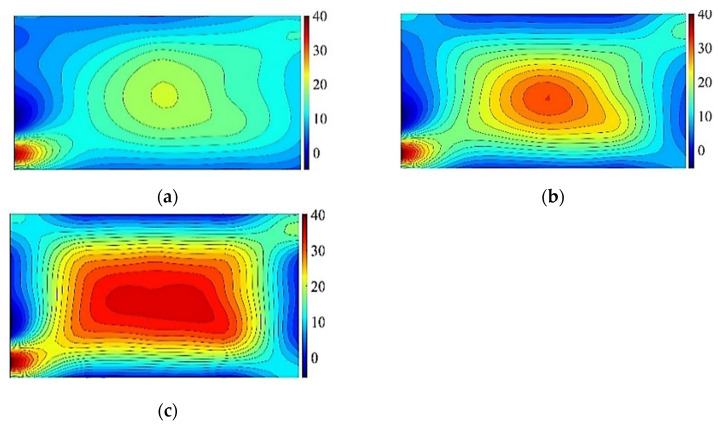
Cold bending and load coupled stress contour plots of TSG-7. (**a**) 1 kPa; (**b**) 3 kPa; (**c**) 5 kPa.

Similarly, according to the data in Table 6, the relationship curve between the *K* value of each influencing factor and its horizontal value at measuring point No. 13 can be drawn. As shown in Figure 9, the influence of the cold bending curvature on the maximum stress increases also almost linearly. The influence of the glass pane thickness on the maximum stress is a slightly nonlinear decrease because the greater the thickness of the glass panes is, the greater the bearing capacity. The effect of the interlayer thickness decreases slowly because of the same effect on the cold bending stress. Figure 9 and Figure 10 are the contour plots of TSG-3 and TSG-7 under loads of 1 kPa, 3 kPa and 5 kPa, and the stress contour plots of the other specimens are basically the same. From the figures, it can be seen that the change process after the coupling of the cold bending stress and load stress, the maximum stress produced by the load is located in the center of the pane, which is different from the position of the maximum cold bending stress. With increasing load, the maximum main tensile stress in the middle of the pane increases gradually, and the range of the maximum stress is gradually expanded from the center of the plate to the surroundings. The stress contour plot gradually becomes close to the characteristics of the uniform load of the general elastic thin plate and exceeds the maximum cold bending main tensile stress at the corner. The stress distribution of the maximum cold bending stress has little change. When the load of TSG-3 reaches the test load of 5 kPa, the stress distribution of the panes is basically consistent with that of the elastic thin plate, but TSG-7 in Figure 11 is slightly different. When the load reaches the test load, the central stress does not exceed the maximum cold bending principal tensile stress at the corner. The other stress distribution characteristics are the same as those of TSG-3.

### 4.2. Effect of the Cold Bending Stress on the Load Stress

**Figure 12:**
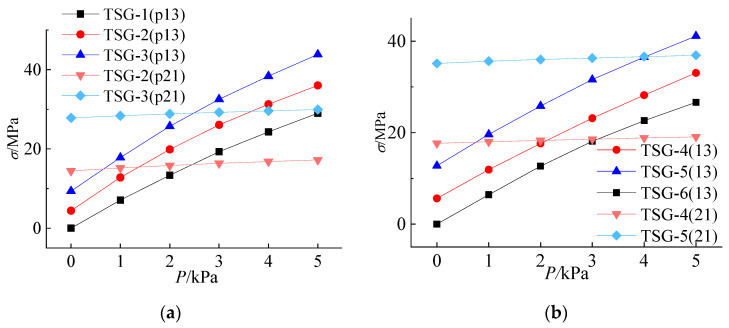

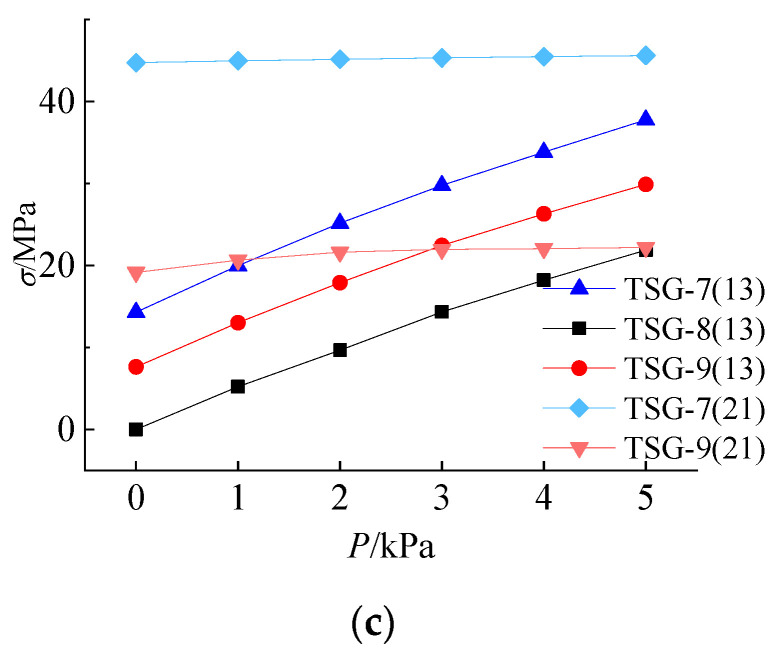
Relationship between the load and the stress. (**a**) t = 5 mm; (**b**) t = 6 mm; (**c**) t = 8 mm.

Based on the analysis of the previous 4.1, the thickness of the interlayer has little effect on the stress of the later load. To analyze the stress variation process of cold-formed glass panes under a load and ignore the influence of the interlayer thickness, the stress variation curves of measuring points No. 13 and No. 21 of each specimen are drawn according to the thickness of the glass panes, as shown in Figure 12. In this figure, the brackets contain the number of stress measuring points. Among them, TGS–1, TGS–6 and TGS–8 are the load stress curves of measuring point No. 13 of the noncold-formed specimen, which are the bottom curves with a starting point of 0. The curves of the other specimens have cold bending stress. Because of the different cold bending stresses of each specimen, the starting point of the curve is also different. Figure 12 shows that the stress growth rate at the No. 13 measuring points in the center of the pane is significantly larger than that at the No. 21 measuring points, and the stress increase process at the center of the pane has a slight nonlinear characteristic. Since glass is a linear elastic material and the laminated material PVB adhesive is a viscoelastic material, it can be judged that the slight nonlinear behavior of the stress increase process is caused by the laminated material. From the curve shape, it can be considered that the effect is not obvious.

By comparing Figure 11a–c, it can be found that the load stress curves at the same measuring point in each figure are almost parallel, which indicates that the load stress relationship is basically the same under the same thickness of the glass panes. Among the curves at the No. 13 measuring points, the bottom curve is the curve of the specimen without cold bending. The two curves above are the curves with the initial cold-formed stress. These two curves are almost parallel to the noncold-formed curve, which indicates that in the loading process, the stress increase law of the specimen with the initial cold-formed stress is basically the same as that of the noncold-formed specimen. Therefore, it can be judged that the load stress relationship of the cold-formed laminated glass panes is basically not affected by the cold-formed stress, and its change law is the same as that of the noncold-formed laminated glass panes. In Figure 11a,b, with an increasing load, the stress of the No. 13 measuring points of the same specimen increases rapidly and intersects with the curve of the No. 21 measuring points, which indicates that the bearing capacity is ultimately controlled by the central point of the panes. However, the TGS–7 in Figure 12c is different. When loaded to the maximum test load, the stress of measuring point No. 13 does not intersect with the curve of measuring point No. 21, which indicates that the bearing capacity is always controlled by the cold bending stress.

## 5. Load Deflection Test Results and Analysis

### 5.1. Test Results on the Load Deflection

**Figure 13:**
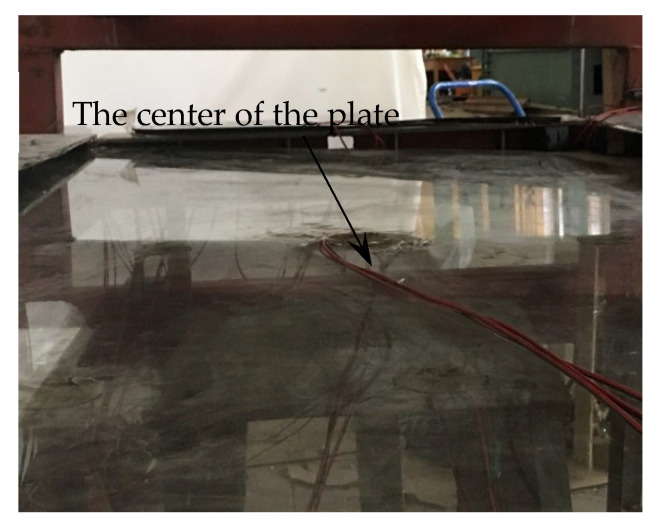
Shape of the glass pane after cold bending.

For the four-sided supporting plate under a uniform load, the center of the plate is the position where the maximum deflection occurs. During the load test, the actual deflection of the center of the pane is obtained by observing the displacement of the No. 5 measuring point in the center of the panes and eliminating the influence of the displacement of the supporting edge; then, superimposed with the cold bending displacement, the deflection of the central point of the glass panes under the coupling effect of the load and cold bending is obtained. The deflection *ɷ* value of the load at all levels is listed in Table 7. The variable *ɷ*_0_ in the table is the displacement of measuring point No. 5 of the pane center after cold bending is completed. The negative value is upward. When the single corner point is cold bending upward, the central point of the pane also produces upward displacement. For example, Figure 13 shows a morphological photograph of the glass panes after the completion of TSG-3 cold bending. From the figure, it can be seen that the deformation of the middle of the panes is upward. The maximum cold bending displacement of the center of the panes in Table 7 is −10.62 mm, which is mainly affected by the cold bending curvature.

### 5.2. Analysis of the Factors That Affect the Load Deflection

To analyze the influence degree of each test factor on the deflection under the load, the range analysis of the maximum deflection of each specimen in Table 7 is conducted according to the method of 3.2. The analysis results are shown in Table 8. It can be seen from the table that the thickness of the glass panes has the greatest influence on the deflection of the load, and the influence of the cold bending curvature is also relatively large, but from the range size, the influence degree of the two is basically the same, and the influence of the thickness of the interlayer is only 3.46% of the thickness, which is relatively small compared with the other two factors and can be ignored.

**Figure 14:**
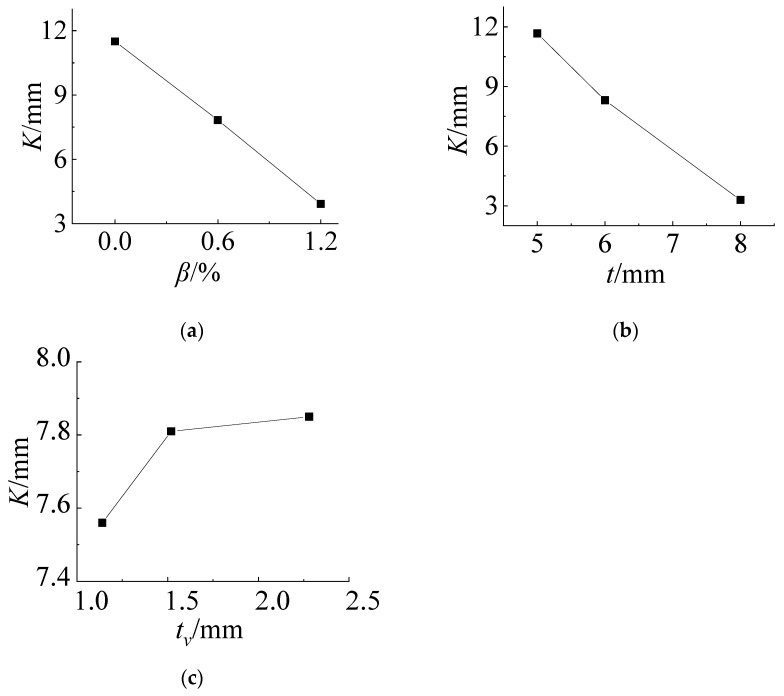
Influence of the test factors on the load deflection. (**a**) *β*~*K*; (**b**) t~*K*; (**c**) *t*_v_~*K*.

The relationship between the value of each influencing factor and its *K* value is drawn, and Figure 14 is obtained. The influence of the cold bending curvature shows a linear downward trend because the larger the cold bending curvature is, the larger the upward displacement of the glass panes, and the smaller the deflection after coupling with the load. The thickness of the glass panes also has the same characteristics because the greater the thickness is, the greater the stiffness. The influence of the interlayer thickness is nonlinear. The greater the thickness is, the greater the deflection. At this time, because the strength of the PVB interlayer material is low, the greater the thickness is, the smaller the combined stiffness of the upper and lower two glass panes is, and it has the characteristics of fast first and slow later. From Table 8, when the interlayer thickness changes from 1.14 mm to 1.52 mm, the *K* value of the deflection increases by 0.28 mm and increases by only 0.01 mm from 1.52 mm to 2.28 mm. When the interlayer thickness is greater than 1.52 mm, it has little influence on the deflection after the cold bending load coupling.

### 5.3. Effect of the Cold Bending Displacement on the Load Deflection

**Figure 15:**
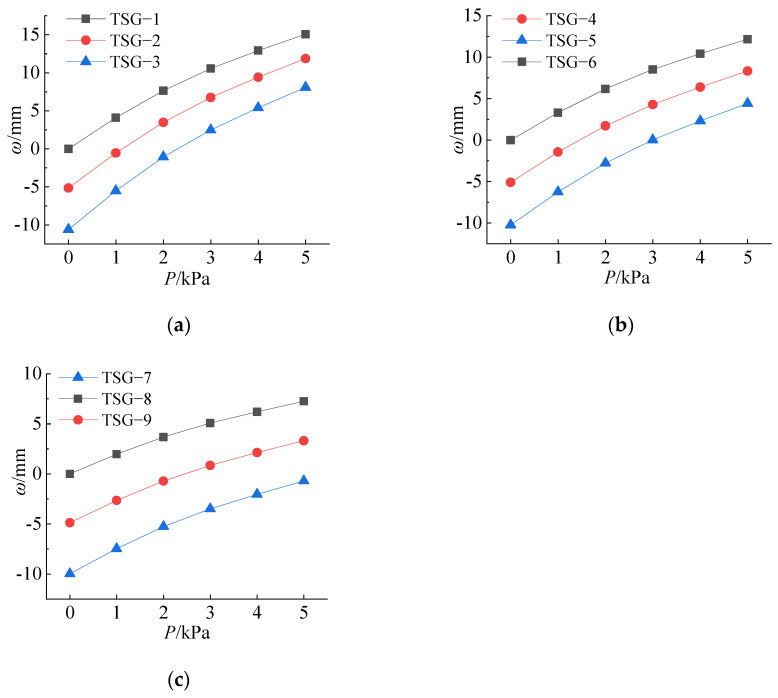
Relationship between the center deflection and the load of the glass panes. (**a**) t = 5 mm; (**b**) t = 6 mm; (**c**) t = 8 mm.

The cold bending displacement is the initial displacement of the late load, and its stress problem belongs to the stress problem of the thin plate with initial displacement. According to the analysis of 5.2, the influence of the interlayer thickness is small. To analyze the influence of the initial displacement on the late load deflection and to ignore the influence of the interlayer thickness according to the method of 4.2, the data in Table 7 are divided into groups according to the thickness of the panes to draw the deflection and load relationship diagram. The curves of the glass pane thicknesses of 5 mm, 6 mm and 8 mm are shown in Figure 15a–c. The load deflection relationship is also slightly nonlinear. The uppermost curves in the three figures are all noncold-bending specimens, and the descending order is the specimens with a cold bending curvature of 0.6% and 1.2%, and the three curves in each figure are almost parallel. Therefore, the deflection change process of the center of the pane caused by the load is similar to the stress change process of the previous 4.2; in other words, the initial deflection of the cold bending has no effect on the deflection change process of the later load. When calculating the maximum deflection, the cold bending deflection and load deflection can also be calculated, and then, the simplified calculation method of superposition can be used.

## 6. Finite Element Analysis

### 6.1. Finite Element Modeling

In this paper, the finite element software ABAQUS is used to simulate glass and cold bending forced placement device with Solid 3D Solid element, and the constitutive model is selected as Mooey–Rivlin’s hyperelastic material to simulate the interlayer PVB glue and rubber gaskets. The value of material constants is as follows: E = 7.2 × 10^5^ N/m^2^, Poisson’s ratio *μ* is 0.2, and the rest is shown in Table 9.

**Figure 16:**
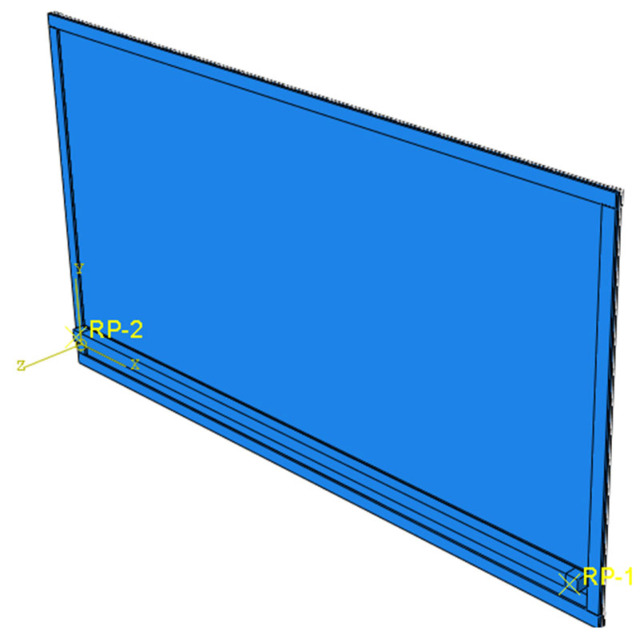
Assembly drawing.

The finite element model is shown in Figure 16.

In the calculation model, the glass panel is the same as the specimen. The glass side is applied translational constraints in the X and Z directions, and the side is applied translational constraints in the Y and Z directions, so that the effect of simple support on the four sides of the laminated glass is realized.

### 6.2. Finite Element Results

#### 6.2.1. Cold Bending Test Results

The simulated stress values *σ*_s_ at NO. 21 of each specimen in the cold bending test and the test stress values *σ*_t_ were listed in Table 10 for comparison.

It can be seen from Table 10 that there is little difference between the stress value of each measuring point and the simulated value. The relative error is generally around −5.00%, and the maximum relative error is −6.52%, which is within the acceptable range. It can be seen from the comparison value and simulated value and experiment value is generally larger than analog values slightly. This is mainly because the actual test device itself has certain defects. The test boundary conditions are not exactly the same as the simulation boundary conditions, and the test itself also has certain errors. But in general, the actual test results are in good agreement with the simulation results, which verifies the feasibility of the simulation, and also indicates that the material property parameters are generally consistent with the actual situation.

**Figure 17:**
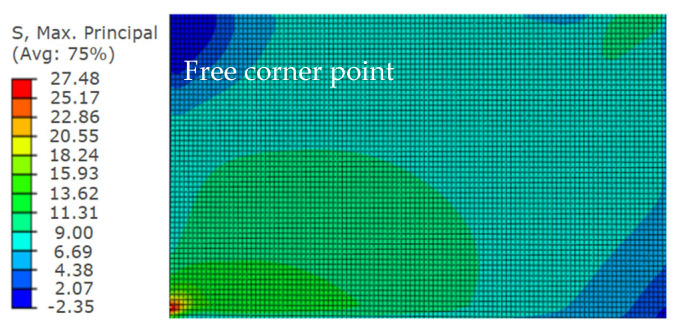
The stress contour plot of TSG-3.

To show the stress distribution, the stress contour plots of tensile surface by ABAQUS calculation results is intercepted. Taking TSG-3 as an example, the calculation results of the other specimens are basically the same, as shown in Figure 17.

#### 6.2.2. Results of Uniformly Distributed Load Test

The simulated stress values at measuring point NO. 13 of each specimen under uniform distributed load of 5 kPa were listed in Table 11 for comparison with the test values. It can be seen from Table 11 that the relative error is about 5%, which is consistent with the actual situation. The relative error of test value and simulation value of uniform load is similar to that of cold bending process. In the loading process of each stage of uniform load, the test value and simulation value are still basically consistent, which verifies the feasibility of this model to simulate uniform load again.

**Figure 18:**
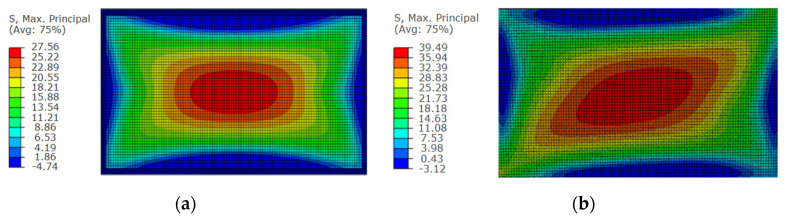
The stress contour plots of tensile surface. (**a**) TSG-1; (**b**) TSG-3.

The stress contour plots of the tensile surface of each specimen under uniform load is basically similar. TSG-1 is selected as the representative of the flat glass, and TSG-3 is selected as the representative of the cold-formed glass, as shown in Figure 18. It can be seen from the figure that the stress gradually decreases from the central area to the surrounding area. The stress on the diagonal of the adjacent corner in the short side direction of the cold bending corner of the cold bending glass is relatively large and goes in a diagonal direction, which is consistent with the stress contour plots of the test value. The diagonal direction of the glass with zero distortion rate is uniform and symmetrical tensile stress. At the same time, the compressive stress is generated in the midpoint area of the four edges of each specimen, which is basically consistent with the experimental results.

## 7. Conclusions

1. The cold bending stress of PVB laminated tempered glass panes with single corner cold bending has a saddle-shaped distribution. The maximum stress appears near the corner point in the short edge direction adjacent to the cold bending single corner, which is located at the edge of the pane and is unfavorable to the stress of the glass pane.
2. The influence of the cold bending curvature on the cold bending stress is the largest, followed by the thickness of the glass panes, and the influence of the interlayer thickness is small. The maximum cold bending stress increases linearly with increasing cold bending curvature. The greater the thickness of the glass panes, the greater the cold bending stress, which is a nonlinear relationship that is first fast and then slow. The greater the thickness of the interlayer is, the smaller the cold bending stress, which is also a nonlinear relationship that is first fast and then slow. The influence of the thickness of the PVB interlayer film is approximately 10% of the cold bending curvature. The influence of the interlayer should be considered when the cold bending stress is unfavorable to the bearing capacity.
3. When the cold bending curvature is large and the glass thickness is large and the interlayer thickness is small, the bearing capacity of the glass panes is controlled by the cold bending stress. Therefore, the cold bending curvature should be limited.
4. When there is single angle warping cold bending, the maximum value of the load stress coupled with the cold bending stress is located in the center of the glass pane. Different from the maximum value of the cold bending stress, the load stress at the maximum value of the cold bending stress increases slowly.
5. The load stress curve and load deflection curve of the cold-formed glass panes show a slight nonlinear relationship under the later load.
6. The thickness of the PVB interlayer has little effect on the change in the stress and the deflection equivalent effects of cold bending and load coupling. When the thickness of the PVB interlayer is beneficial to the bearing capacity, its effect can be ignored.

## Figures and Tables

**Table 1:** Test parameters.

Specimen	*a* × *b*/mm	t/mm	*t*_v_/mm	*β*_max_/%	*s*/mm
TSG-1	2200 × 1200	5	1.14	0.0	0
TSG-2	2200 × 1200	5	1.52	0.6	30
TSG-3	2200 × 1200	5	2.28	1.2	60
TSG-4	2200 × 1200	6	1.14	0.6	30
TSG-5	2200 × 1200	6	1.52	1.2	60
TSG-6	2200 × 1200	6	2.28	0.0	0
TSG-7	2200 × 1200	8	1.14	1.2	60
TSG-8	2200 × 1200	8	1.52	0.0	0
TSG-9	2200 × 1200	8	2.28	0.6	30

**Table 2:** The stress results of the cold bending test.

Specimen	t/mm	*t*_v_/mm	*β*/%	*σ*_m_/MPa	
				NO.13	NO.21
TSG-1	5	1.14	0.0	0.00	0.00
TSG-2	5	1.52	0.6	4.42	14.45
TSG-3	5	2.28	1.2	9.36	28.85
TSG-4	6	1.14	0.6	5.62	17.66
TSG-5	6	1.52	1.2	12.76	35.13
TSG-6	6	2.28	0.0	0.00	0.00
TSG-7	8	1.14	1.2	14.29	44.25
TSG-8	8	1.52	0.0	0.00	0.00
TSG-9	8	2.28	0.6	7.63	19.16

**Table 3:** Range analysis of the cold bending stress at No. 21.

*K*	Influencing Factors		
	t	*t*_v_	*β*
*K*_1_/MPa	14.43	20.64	0.00
*K*_2_/MPa	17.60	16.53	17.09
*K*_3_/MPa	21.14	16.00	36.08
Range *R*/MPa	6.71	4.64	36.08
Sequence	2	3	1
Ratio/%	18.6	12.9	1.00

**Table 4:** Range analysis of the cold bending stress at No. 13.

*K*	Influencing Factors		
	t	*t*_v_	*β*
*K*_1_/MPa	4.59	6.64	0.00
*K*_2_/MPa	6.13	5.73	5.89
*K*_3_/MPa	7.31	5.66	12.14
Range *R*/MPa	2.72	0.98	12.14
Sequence	2	3	1
Ratio/%	22.41	8.07	1.00

**Table 5:** The stress results of the load test.

Specimen	t/mm	*t*_v_/mm	*β*/%	*σ*_m_/MPa	
				NO.13	NO.21
TSG-1	5	1.14	0.0	29.02	10.03
TSG-2	5	1.52	0.6	36.02	17.23
TSG-3	5	2.28	1.2	43.84	29.93
TSG-4	6	1.14	0.6	33.06	19.08
TSG-5	6	1.52	1.2	41.11	36.91
TSG-6	6	2.28	0.0	26.62	8.34
TSG-7	8	1.14	1.2	37.76	45.61
TSG-8	8	1.52	0.0	21.87	6.15
TSG-9	8	2.28	0.6	27.89	22.19

**Table 6:** Range analysis of the influencing factors of the load stress at No. 13.

*K*	Influencing Factors		
	t	*t*_v_	*β*
*K*_1_/MPa	36.29	33.28	25.84
*K*_2_/MPa	33.60	33.00	32.32
*K*_3_/MPa	29.17	32.78	40.90
Range *R*/MPa	7.12	0.5	15.06
Sequence	2	3	1
Ratio/%	47.2	3.32	1.00

**Table 7:** Deflection test results of the pane’s center.

Specimen	*ɷ*_0_/mm	*ɷ*/mm				
		1 kPa	2 kPa	3 kPa	4 kPa	5 kPa
TSG-1	0.00	4.09	7.65	10.56	12.91	15.07
TSG-2	−5.15	−0.54	3.48	6.76	9.42	11.86
TSG-3	−10.62	−5.53	−1.08	2.47	5.40	8.08
TSG-4	−5.09	−1.43	1.72	4.30	6.39	8.33
TSG-5	−10.23	−6.24	−2.78	0.03	2.32	4.42
TSG-6	0.00	3.31	6.18	8.52	10.42	12.17
TSG-7	−9.98	−7.47	−5.25	−3.48	−2.03	−0.70
TSG-8	0.00	1.98	3.68	5.08	6.21	7.26
TSG-9	−4.87	−2.64	−0.71	0.86	2.14	3.32

**Table 8:** Range analysis of the factors that influence the load deflection.

*K*	Influencing Factors		
	t	*t*_v_	*β*
*K*_1_/MPa	11.67	7.57	11.50
*K*_2_/MPa	8.31	7.85	7.84
*K*_3_/MPa	3.29	7.86	3.93
Range *R*/MPa	8.38	0.29	7.57
Sequence	1	3	2
Ratio/%	1	3.46	90.33

**Table 9:** The material properties.

Materials	E (N/mm^2^)	*μ*	*ρ* (T/m^3^)
Glass	72,000	0.20	2.56
Steel	206,000	0.20	7.85
PVB	/	0.49	1.07
Rubber gasket	/	0.49	1.10

**Table 10:** The stress results at NO. 21.

Specimen	*σ*_s_/MPa	*σ*_t_/MPa	Relative Error/%
TSG-2	14.45	13.61	−5.81%
TSG-3	28.85	27.48	−4.47%
TSG-4	17.66	16.87	−4.47%
TSG-5	35.13	32.84	−6.52%
TSG-7	44.25	41.75	−5.65%
TSG-9	19.16	18.33	−4.32%

**Table 11:** The stress results at NO. 13.

Specimen	*σ*_s_/MPa	*σ*_t_/MPa	Relative Error/%
TSG-1	29.02	27.56	−5.03%
TSG-2	36.02	34.47	−4.30%
TSG-3	43.84	41.65	−4.90%
TSG-4	33.06	31.54	−4.60%
TSG-5	41.11	39.48	−3.96%
TSG-6	26.62	24.95	−6.27%
TSG-7	37.76	36.71	−2.78%
TSG-8	21.87	20.67	−5.49%
TSG-9	27.89	26.34	−5.50%

## Data Availability

Not applicable.
